# Thyroid hormones and carnitine in the second trimester negatively affect neonate birth weight: A prospective cohort study

**DOI:** 10.3389/fendo.2023.1080969

**Published:** 2023-02-21

**Authors:** Mengmeng Yang, Man Sun, Chenyu Jiang, Qianqian Wu, Ying Jiang, Jian Xu, Qiong Luo

**Affiliations:** ^1^ Women’s Hospital of Zhejiang University School of Medicine, Hangzhou, China; ^2^ The Fourth Affiliated Hospital of Zhejiang University School of Medicine, Yiwu, China

**Keywords:** free carnitine, thyroid hormones, neonate birthweight, the second trimester, gestation

## Abstract

**Background:**

Maternal thyroid hormones and carnitine are reported to affect neonate birth weight during the second trimester, which is one of the most important markers for fetal growth and perinatal mortality and morbidity. Nevertheless, the effect of thyroid hormone and carnitine in the second trimester on birth weight has yet to be understood.

**Method:**

This was a prospective cohort study with 844 subjects enrolled during the first trimester. Thyroid hormones, free carnitine (C0), neonate birth weight, as well as other related clinical and metabolic data were collected and assessed.

**Results:**

Pre-pregnancy weight and body mass index (BMI) as well as neonate birth weight were significantly different among different free thyroxine (FT4) level groups. Maternal weight gain and neonate birth weight varied significantly when grouped by different thyroid-stimulating hormone (TSH) levels. There was a significantly positive correlation between C0 and TSH (r = 0.31), free triiodothyronine (FT3) (r = 0.37), and FT4 (r = 0.59) (all P < 0.001). In addition, a significantly negative influence was found between birth weight and TSH (r = −0.48, P = 0.028), so as C0 (r = −0.55, P < 0.001) and FT4 (r = −0.64, P < 0.001). Further assessment detected a stronger combined effect of C0 and FT4 (P < 0.001) and of C0 and FT3 (P = 0.022) on birth weight.

**Conclusion:**

Maternal C0 and thyroid hormones are of great importance in neonate birth weight, and routine examination of C0 and thyroid hormones during the second trimester has a positive effect on the intervention of birth weight.

## 1 Introduction

Adequate thyroid hormones (THs) are crucial for the fetal growth and metabolism and play an important role in neurodevelopment (1, 2). The fetal thyroid grand starts to develop at 12th week of gestation and is functionally mature around the 18th to 20th week, whereas, for the first half of pregnancy, the fetus relies entirely on the supply of maternal TH through the transplacental passage (2–4). Abnormal maternal thyroid function during pregnancy is associated with adverse obstetrical and offspring outcomes, such as spontaneous abortion, anemia, preeclampsia, placental abruption, congenital anomalies, preterm birth and/or low birth weight, fetal distress in labor, stillbirth and/or perinatal death, and postpartum hemorrhage (3).

Carnitine exists as free carnitine (C0) and acylcarnitine fractions in blood and plays an important role in fatty acid oxidation during the gestational metabolism (4). Carnitine mainly comes from food, especially red meat, fish, and dairy products (5). Carnitine is critical for the transfer of activated long-chain fatty acids from the cytoplasm to the mitochondria for β-oxidation, resulting in the esterification of carnitine to form acylcarnitine derivatives (6). Some evidence suggests that carnitine deficiency is manifested in gestational diabetes mellitus (GDM), leading to the development of macrosomia and small for gestational age (SGA). Clinical studies showed that applying C0 (1 g/day) for a few weeks could relieve hyperthyroidism symptoms in patients. Carnitine was hypothesized to act in the periphery by antagonizing TH action. However, the correlation between C0 and THs in mid-pregnancy remains unknown.

Birth weight is one of the most important markers for fetal growth and development *in utero*, which reflects fetal adaptations to the intrauterine environment. SGA newborns have an increased risk of prenatal mortality (7), whereas large for gestational age (LGA) newborns have a higher risk of obesity and diabetes mellitus in later life (2). Various studies have shown that a higher maternal free thyroxine (FT4) level is associated with a lower birth weight (3, 8, 9). However, studies undertaken so far provide conflicting evidence concerning the impact of C0 on TH and birth weight.

In the present study, we investigated the associations between maternal serum thyroid parameters and carnitine-related metabolites during the second trimester of pregnancy. Furthermore, we examined whether the birth weight was modified by maternal serum TH and carnitine.

## 2 Material and methods

### 2.1 Study subjects

This was a prospective cohort study. Pregnant women who received regular perinatal healthcare in the Outpatient Department of the Women’s Hospital School of Medicine Zhejiang University and delivered in the hospital between June 2017 and April 2019 were recruited. A total of 844 pregnant women with complete demographic and obstetric data were included for analysis. This study was approved by the Ethics Committee of Women’s Hospital School of Medicine Zhejiang University.

All pregnant women were enrolled during the first trimester, all of whom had measured thyroid-stimulating hormone (TSH), FT4, free triiodothyronine (FT3), and total thyroxine (TT4) concentration in the second trimester, and neonatal birth weight data were available. Women with multiple pregnancies, accompanied with pregnancy complications such as abortion, GDM, and hypertensive disorders, using medication known to interfere thyroid hormones, or had a history of thyroid diseases were excluded.

Maternal clinical characteristics—including age, height, pre-pregnancy and prenatal weight and body mass index (BMI), gravity, parity, mode of delivery, educational level, and neonatal birth weight—were obtained from hospital information system and child care system.

The blood samples were collected after overnight fasting, and samples were centrifuged within 6 h. The concentrations of TSH, FT4, FT3, and TT4 were determined according to the measurement instructions.

### 2.2 Metabolic profiling detection by LC-MS/MS

We aim to investigate the 31-carnitine-related plasma metabolite level of pregnant women at the second trimester. We also obtain their neonate blood plasma sample. The blood samples were stored at −20°. Furthermore, the samples were prepared by tandem mass spectrometry (4000 QTrapTM; AB Sciex, Darmstadt, Germany) to test the concentration. The method used in the present study was essentially a modification of the procedure described elsewhere. Amino acid (AA) and acylarnitine (AC) were quantified using appropriate isotope-labeled standards. Liquid Chromatogram (LC) separation was performed on an Acquity UPLC HSS T3 column (2.1 × 100 mm, 100A°, 1.8-µm particle size; Waters Corporation, MA) using water with 0.1% formic acid (0.1% methanol and 5 mM ammonium acetate) detected with a Xevo-G2-QTOF MS (Waters Corporation) operating in a positive mode. Raw data were processed using TargetLynx as described previously. Accuracy of quantification was below 6% for all quantified metabolites except glutamic acid (13.9%). Quantitative data were obtained using MetIDQTM software.

### 2.3 Statistical analysis

The data were expressed as mean ± standard deviation (SD). The baseline characteristics of the subjects were described, and the p-values are indicated. Binary variables were presented as frequency and percentage and were compared using the Chi-squared test. A nonparametric test or a t-test was used to compare the medians of continuous variables. The heatmap was available from the package “ggplot” as an enhanced version or its basic function stats in R. We used the liner regression model, as well as multiple regressions to investigate the association of C0, FT3, FT4, TSH, and TT4 with birth weight. We assessed the combined effects of C0 and FT4 on birth weight by adding a product interaction term of the C0 × FT4 to the model. The same analysis was done on the effect of other hormone on birth weight. A heatmap was constructed to display the differences in birth weight. All statistical analyses were performed using R statistical software version 3.4.1 (package rms, ggplot, visreg, and mass) or Statistical Package of Social Sciences version 20.0 for Windows (IBM Corp., Armonk, NY). In all analyses, *P* < 0.05 was considered statistically significant.

## 3 Results

### 3.1 Clinical characteristics of subjects grouped by thyroid hormone

The maternal characteristics grouped by FT4 quartile are shown in **Table 1**. The different ranges of FT4 are as follows: Q1: 8.33–9.72 pmol/L; Q2: 9.73–10.65 pmol/L; Q3: 10.66–11.57 pmol/L; and Q4: 11.58–15.06 pmol/L. We found that pre-pregnancy weight and BMI as well as weight gain were significantly different among the four groups with a higher level in the lower FT4–level group. We also found that compared with the higher FT4–level group, birth weight was significantly heavier in the lower groups. There were significantly statistically differences in height and gestational week at delivery among different groups, but no difference in maternal age, nulliparous rate, and ART rate among these four groups.

**Table 1:** Clinical characteristics of pregnancy by FT4 quartile.

FT4 groups (pmol/L)	Q1 (n = 211)	Q2 (n = 211)	Q3 (n = 211)	Q4 (n = 211)	*P*
	8.33–9.72	9.73–10.65	10.66–11.57	11.58–15.06	
**Maternal age (years)**	31.48 ± 2.42	31.28 ± 2.62	31.65 ± 3.05	31.07 ± 2.79	0.149
**Height (cm)**	162.87 ± 5.49	159.65 ± 5.14	162.63 ± 2.27	160.44 ± 1.98	**<0.001**
**Pre-pregnancy weight (kg)**	65.58 ± 4.83	64.52 ± 3.91	63.48 ± 3.65	64.18 ± 2.97	**<0.001**
**Pre-pregnancy BMI (kg/m^2^)**	22.65 ± 4.01	21.86 ± 3.74	21.24 ± 3.19	21.74 ± 3.26	**<0.001**
**Weight gain (kg)**	22.38 ± 4.21	21.65 ± 4.01	20.88 ± 2.45	19.78 ± 2.67	**<0.001**
**Gestational week at delivery (weeks)**	38.3 ± 0.67	38.4 ± 0.78	39.4 ± 0.72	38.7 ± 0.87	**<0.001**
**Nulliparous (%)**	166 (78.7)	164 (77.7)	167 (79.1)	165 (78.2)	0.987
**ART (%)**	13 (6.2)	12 (5.7)	9 (4.3)	12 (5.7)	0.843
**Birth weight (g)**	3633.23 ± 99.78	3527.79 ± 86.63	3404.56 ± 100.45	3217.93 ± 77.94	**<0.001**

Bold values are presented as mean ± SD or number (%).

FT4, free thyroxine; BMI, body mass index; ART, artificial reproductive technology.

**Table 2** presents clinical and biochemical characteristics of participants grouped by TSH. In the group with TSH < 2.5 mIU/L, pre-pregnancy BMI, weight gain, and birth weight were significantly higher; whereas, in the higher TSH–level group, pre-pregnancy weight and gestational week at delivery were higher. There was no difference in maternal age, nulliparous rate, and ART rate between the two groups.

**Table 2:** Clinical and biochemical characteristics of pregnancy by TSH.

TSH groups (mIU/L)	TSH<2.5 (n = 762)	TSH ≥ 2.5 (n = 82)	*P*
**Maternal age (years)**	31.18 ± 2.42	31.65 ± 2.05	0.091
**Height (m)**	163.27 ± 5.49	162.63 ± 3.72	0.3032
**Pre-pregnancy weight (kg)**	63.58 ± 4.83	65.48 ± 3.65	**0.001**
**Pre-pregnancy BMI (kg/m^2^)**	22.65 ± 4.01	21.24 ± 3.19	**0.002**
**Weight gain (kg)**	21.38 ± 4.21	18.23 ± 2.45	**<0.001**
**Gestational week at delivery (weeks)**	38.3 ± 0.67	39.4 ± 0.72	**<0.001**
**Nulliparous (%)**	674 (88.5)	73 (89.02)	0.877
**ART (%)**	73 (9.6)	4 (4.9)	0.160
**Birth weight (g)**	3543.23 ± 99.78	3304.56 ± 100.45	**<0.001**

Bold values are presented as mean ± SD or number (%).

TSH, thyroid-stimulating hormone; BMI, body mass index; ART, artificial reproductive technology.

### 3.2 Biochemical characteristics of subjects grouped by thyroid hormone

Among 31 carnitine-related metabolites, we selected the metabolites with statistically significant differences and listed them in **Table 3**. Our results showed that carnitine-related AA [alanine (ALA), tyrosine (TYR), and valine (VAL)], short-chain AC (C2, C3, C3DC+C4OH, and C5:1), medium-chain AC (C6DC and C12), and long-chain AC (C14, C16:1) were significantly different among the four groups. As the FT4 level increased, ALA, TYR, C14, and C16:1 decreased, whereas VAL, C0, C2, C3, C3DC+C4OH, C5, C6DC, and C12 increased. There were also statistical differences in LEU+ILE+PRO-OH, SA, C4, C8, C14OH, C16, C18, and C18OH among these four groups.

**Table 3:** Carnitine-related metabolites grouped by FT4 quartile (n = 844).

AA and AC profiles (μmol/L)	Q1	Q2	Q3	Q4	*P*
**ALA**	399.64 ± 21.46	368.87 ± 35.12	343.56 ± 24.14	322.77 ± 28.62	**<0.001**
**ARG**	9.16 ± 2.29	8.74 ± 2.94	8.76 ± 1.82	8.81 ± 2.38	0.231
**GLY**	225.64 ± 25.11	226.66 ± 25.32	224.28 ± 18.34	227.45 ± 28.40	0.580
**LEU+ILE+PRO-OH**	135.38 ± 24.42	134.16 ± 31.72	130.85 ± 23.85	139.89 ± 23.96	**0.005**
**MET**	10.47 ± 3.32	10.06 ± 3.15	10.81 ± 2.87	10.42 ± 2.85	0.095
**ORN**	52.63 ± 12.76	52.86 ± 12.16	52.33 ± 11.44	53.37 ± 12.99	0.850
**PHE**	48.76 ± 8.49	48.65 ± 9.84	49.03 ± 8.43	49.93 ± 7.88	0.420
**PRO**	85.74 ± 16.32	86.21 ± 17.71	83.31 ± 14.06	86.34 ± 14.82	0.166
**SA**	0.77 ± 0.14	0.79 ± 0.13	0.78 ± 0.11	0.81 ± 0.12	**0.009**
**TYR**	41.73 ± 9.16	39.73 ± 7.86	37.63 ± 4.65	35.63 ± 4.16	**<0.001**
**VAL**	118.72 ± 17.35	123.72 ± 18.15	125.15 ± 19.27	127.20 ± 29.27	**0.001**
**C0**	16.35 ± 3.39	18.00 ± 2.95	20.51 ± 2.68	22.35 ± 3.62	**<0.001**
**C2**	2.67 ± 0.82	2.86 ± 0.76	3.04 ± 0.78	3.14 ± 0.84	**<0.001**
**C3**	0.30 ± 0.18	0.42 ± 0.14	0.50 ± 0.21	0.60 ± 0.19	**<0.001**
**C3DC+C4OH**	0.23 ± 0.12	0.28 ± 0.11	0.33 ± 0.09	0.45 ± 0.14	**<0.001**
**C4**	0.09 ± 0.01	0.08 ± 0.02	0.09 ± 0.02	0.10 ± 0.02	**<0.001**
**C5**	0.12 ± 0.01	0.23 ± 0.09	0.27 ± 0.10	0.34 ± 0.17	**<0.001**
**C5DC+C6OH**	0.38 ± 0.12	0.36 ± 0.13	0.39 ± 0.11	0.38 ± 0.13	0.085
**C6**	0.018 ± 0.011	0.017 ± 0.012	0.017 ± 0.011	0.018 ± 0.013	0.678
**C6DC**	0.018 ± 0.012	0.21 ± 0.17	0.28 ± 0.11	0.36 ± 0.13	**<0.001**
**C8**	0.50 ± 0.13	0.49 ± 0.18	0.56 ± 0.19	0.54 ± 0.16	**<0.001**
**C8:1**	0.063 ± 0.022	0.060 ± 0.025	0.062 ± 0.028	0.062 ± 0.024	0.654
**C10**	0.056 ± 0.019	0.056 ± 0.022	0.058 ± 0.021	0.059 ± 0.020	0.337
**C12**	0.12 ± 0.182	0.14 ± 0.038	0.16 ± 0.034	0.18 ± 0.056	**<0.001**
**C14**	0.28 ± 0.045	0.26 ± 0.052	0.24 ± 0.048	0.23 ± 0.092	**<0.001**
**C14OH**	0.014 ± 0.0055	0.012 ± 0.0052	0.013 ± 0.0058	0.013 ± 0.0064	**0.005**
**C16**	0.023 ± 0.012	0.026 ± 0.013	0.026 ± 0.011	0.024 ± 0.015	**0.035**
**C16:1**	0.22 ± 0.056	0.20 ± 0.034	0.18 ± 0.067	0.13 ± 0.033	**<0.001**
**C18**	0.57 ± 0.20	0.53 ± 0.22	0.59 ± 0.24	0.56 ± 0.21	**0.040**
**C18:1**	0.55 ± 0.17	0.54 ± 0.18	0.53 ± 0.12	0.56 ± 0.22	0.335
**C18OH**	0.015 ± 0.0047	0.016 ± 0.0043	0.012 ± 0.0069	0.015 ± 0.0026	**<0.001**

Bold values are presented as mean ± SD.

FT4, free thyroxine; AA, amino acid; AC, acylarnitine; ALA, alanine; ARG, arginine; GLY, glycine; LEU, leucine; ILE, isoleucine; MET, methionine; ORN, ornithine; PHE, phenylalanine; PRO, proline; SA, salicylic acid; TYR, tyrosine; VAL, valine.

Carnitine-related metabolites grouped according to the TSH level are shown in **Table 4**. Compared with the lower TSH–level group, ALA and glycine (GLY) significantly decreased in the higher TSH–level group. LEU+ILE+PRO-OH, VAL, C0, C2, C3, C3DC+C4OH, C5, C6DC, C8:1, C10, C12, and C14 were significantly higher when TSH increased. There was a statistical difference in C4 between groups. On the basis of previous studies, C0 has a vital role in metabolism.

**Table 4:** Thirty-one carnitine-related metabolites by TSH (n = 844).

AA and AC profiles (μmol/L)	TSH<2.5	TSH ≥ 2.5	*P*
**ALA**	378.64 ± 35.52	367.89 ± 41.67	**0.011**
**ARG**	8.93 ± 2.52	8.87 ± 3.01	0.841
**GLY**	0.81 ± 0.10	0.64 ± 0.12	**<0.001**
**LEU+ILE+PRO-OH**	35.63 ± 4.16	41.73 ± 9.16	**<0.001**
**MET**	10.56 ± 3.44	10.72 ± 3.56	0.690
**ORN**	53.65 ± 11.64	54.06 ± 12.08	0.763
**PHE**	48.78 ± 6.72	49.23 ± 7.78	0.571
**PRO**	86.34 ± 12.29	85.89 ± 15.89	0.760
**SA**	0.76 ± 0.15	0.77 ± 0.18	0.574
**TYR**	38.87 ± 6.87	39.12 ± 7.08	0.755
**VAL**	118.72 ± 17.35	127.20 ± 29.27	**<0.001**
**C0**	16.35 ± 3.62	20.00 ± 3.39	**<0.001**
**C2**	2.78 ± 0.62	3.41 ± 0.78	**<0.001**
**C3**	0.32 ± 0.18	0.62 ± 0.21	**<0.001**
**C3DC+C4OH**	0.03 ± 0.011	0.05 ± 0.013	**<0.001**
**C4**	0.09 ± 0.02	0.08 ± 0.02	**<0.001**
**C5**	0.02 ± 0.015	0.04 ± 0.014	**<0.001**
**C5DC+C6OH**	0.39 ± 0.13	0.37 ± 0.14	0.189
**C6**	0.017 ± 0.011	0.018 ± 0.013	0.443
**C6DC**	0.32 ± 0.12	0.44 ± 0.124	**<0.001**
**C8**	0.52 ± 0.19	0.50 ± 0.16	0.359
**C8:1**	0.38 ± 0.027	0.45 ± 0.056	**<0.001**
**C10**	0.37 ± 0.062	0.46 ± 0.041	**<0.001**
**C12**	0.31 ± 0.031	0.42 ± 0.023	**<0.001**
**C14**	0.28 ± 0.025	0.33 ± 0.032	**<0.001**
**C14OH**	0.014 ± 0.0054	0.015 ± 0.0048	0.108
**C16**	0.026 ± 0.013	0.025 ± 0.012	0.505
**C16:1**	0.19 ± 0.064	0.20 ± 0.058	0.175
**C18**	0.58 ± 0.19	0.60 ± 0.21	0.370
**C18:1**	0.56 ± 0.17	0.57 ± 0.15	0.609
**C18OH**	0.014 ± 0.0045	0.014 ± 0.0039	0.999

Bold values are presented as mean ± SD.

TSH, thyroid-stimulating hormone; AA, amino acid; AC, acylarnitine; ALA, alanine; ARG, arginine; GLY, glycine; LEU, leucine; ILE, isoleucine; MET, methionine; ORN, ornithine; PHE, phenylalanine; PRO, proline; SA, salicylic acid; TYR, tyrosine; VAL, valine.

### 3.3 A clustering heatmap illustrating the relationship between thyroid hormones and carnitine metabolites

**Figure 1:**
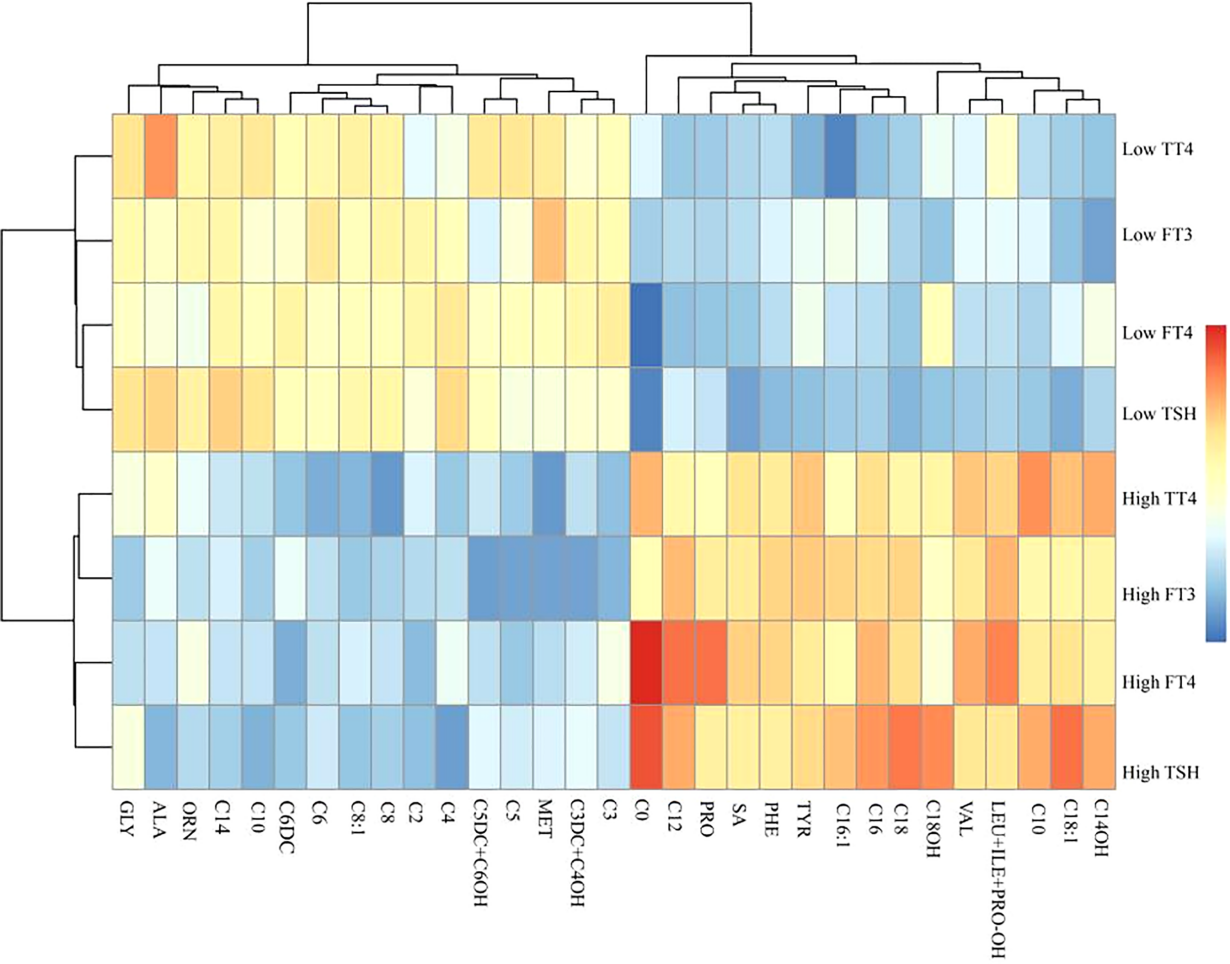
A clustering heatmap illustrating the classification between carnitine-related metabolites and thyroid hormones. Both the rows of thyroid hormones and the columns of carnitine-related metabolites have been clustered, respectively.

We used a clustering heatmap to describe the relationship between THs and 31 carnitine-related metabolites (**Figure 1**). In the row clustering step, pregnant women were grouped according to TH levels. We defined the low TH level and the high TH level as less than 10th centile and as more than 10th centile, respectively, of all participants’ full range. In addition, every biomarker was clustered into different subgroups on the column side according to their color patterns in the center grids of heatmap. Red color indicates a high expression content, and blue color indicates a low expression content. The level of C0 is higher in the high FT4–level and high TSH–level groups. Heatmaps provided a systematic and clustered visualization of the analyzed data, facilitating monitoring of TH levels and carnitine-related metabolites.

### 3.4 Relationship between C0 and TSH, FT3, FT4, and TT4

**Figure 2:**
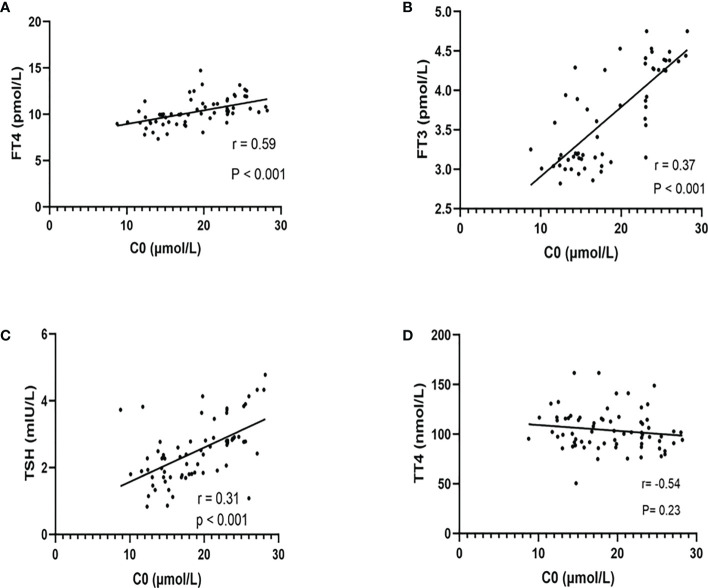
The relationship between free carnitine and thyroid hormones (FT4, FT3, TSH, and TT4). The correlation between C0 and FT4 **(A)**, FT3 **(B)**, TSH **(C)**, and TT4 **(D)** was shown above. There were significant positive correlations between C0 and FT4, FT3, and TSH rather than TT4. r = Spearman’s correlation coefficient.

The relationships between C0 and TSH, FT3, FT4, and TT4 of all pregnant women are shown in **Figure 2**. We observed that C0 was positively correlated with TSH (r = 0.31, *P* < 0.001), FT3 (r = 0.37, *P* < 0.001), and FT4 (r = 0.59, *P* < 0.001). There was no significant correlation between C0 and TT4.

### 3.5 Effects of C0, TSH, FT3, FT4, and TT4 on birth weight

**Figure 3:**
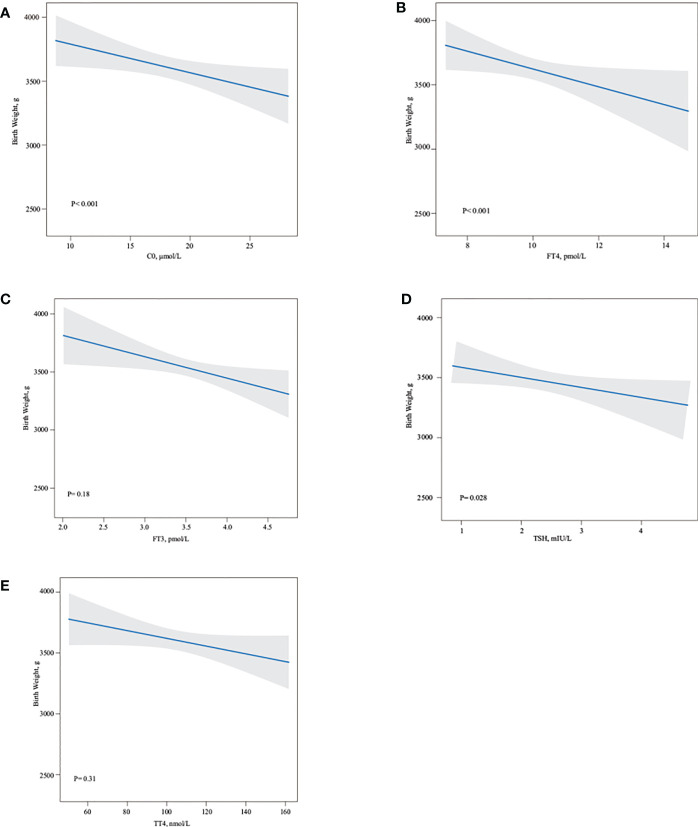
Association of maternal free carnitine and thyroid hormones in the second trimester pregnancy and neonate birth weight. Linear regression models for C0 **(A)**, FT4 **(B)**, FT3 **(C)**, TSH **(D)**, TT4 **(E)** with birth weight, as predicted mean with 95% CI, were shown above. C0, FT4, and TSH negatively influenced neonate birth weight (all *P* < 0.05), whereas there were no statistically association between FT3 and TT4 and neonate birth weight. Analyses were adjusted for maternal age, BMI, parity, and fetal sex.

In the second trimester, a higher TSH (r = −0.48, P = 0.028) level was associated with a lower birth weight (**Figure 3D**). There was also a negative association between C0 (r = −0.55, P < 0.001) and FT4 (r =−0.64, P < 0.001) and birth weight (**Figures 3A, B**). There was no statistically significant difference between TT4 and birth weight (**Figures 3C, E**).

Linear multiple regression model demonstrated that TSH, FT4, and C0 were negatively associated with birth weight. The birth weight decreased by 12.079 g (95% CI: −18.642, −8.532), by 29.203 g (95% CI: −33.149, −15.511), and by 21.079 g (95% CI: −29.842, −13.316) for every unit increase in C0, FT4, and TSH concentrations, respectively (**Table 5**).

**Table 5:** Multivariable analysis of thyroid hormone, C0 in relation to birth weight.

Variables	β coefficient	P-value
**TSH**	**−21.079 (−29.842, −13.316)**	**0.013**
**FT4**	**−29.203 (−33.149, −15.511)**	**0.004**
**FT3**	**−22.728 (−37.836, 11.456)**	**0.125**
**TT4**	**−18.185 (−13.947, 36.511)**	**0.403**
**C0**	**−12.079 (−18.642, −8.532)**	**0.038**

Bold values are presented as mean ± SD. TSH, thyroid-stimulating hormone; FT4, free thyroxine; FT3, free triiodothyronine; TT4, total thyroxine; C0, free carnitine.

### 3.6 Combined effects of C0 and TSH, FT3, FT4, or TT4 on birth weight

**Figure 4:**
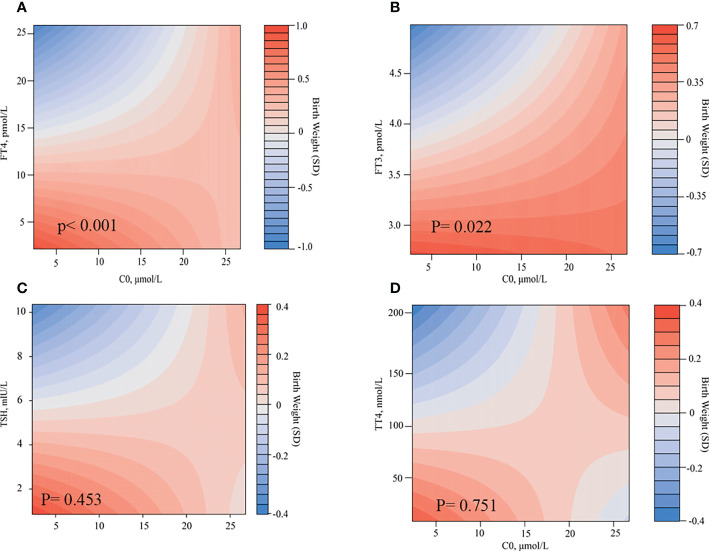
Combined effects of C0 and thyroid hormones in the second trimester on birth weight. Heatmap (filled contour plot) for the correlation of birth weight (red indicates increased gestational age–adjusted birth weight, and blue indicates decreased gestational age–adjusted birth weight) according to the interaction of C0 and FT4 **(A)**, FT3 **(B)**, TSH **(C)**, and TT4 **(D)** in the second trimester. Analyses were adjusted for maternal age, BMI, parity, and fetal sex.

We found that the level of TSH, FT3, FT4, and C0 in the second trimester would have greater effects on birth weight. Meanwhile, we found a relationship between C0 and thyroid function parameters. Therefore, we assessed the combined effects of C0 and THs, including TSH, FT3, FT4, and TT4, on birth weight. **Figure 4** shows the heatmaps (fulfilled contour plot) for the combined association of C0 (x-axis) and TSH, FT4, or FT3 in the second trimester (y-axis) with birth weight (z-axis: red indicates higher birth weight, and blue indicates lower birth weight). For the low level of C0, the birth weight will be decreased, with a gradual increase in the FT4/FT3 level. For the high level of C0, the effect on birth weight was more obvious. When carnitine is increased to an appropriate level and the thyroid concentration is high (FT4: 18.4 pmol/L; C0: 17.5 µmol/L; FT3: 4.3 pmol/L; and C0: 16.3 µmol/L), the birth weight would be at the average level. When the FT4//FT3 level is high, even if the carnitine level decreases, the birth weight may remain at the present level. Free thyroxine (FT4/FT3) may play a greater role in regulating birth weight than C0. There was a considerable difference according the combination of FT4 and C0 in the second trimester (*P* < 0.001, **Figure 4A**). Lower FT4 and C0 levels were associated with a 0.8 SD higher birth weight. A lower FT4 level but a median C0 level had an influence of a 0.5 SD higher birth weight. A low FT4 level and a higher C0 level had no effect on birth weight. A low FT3 level and a low C0 level were associated with a 0.35 SD higher birth weight. A low FT3 level but a median C0 level were associated with a 0.2 SD higher birth weight. A low FT3 level and a higher C0 level had no effect on birth weight. In line with these analyses, a low FT4 level and a low C0 level were associated with more pronounced effects on birth weight. However, the effects estimate of birth weight did not different when combinations of TSH and C0, TT4, and C0.

## 4 Discussion

Our research showed that there was a significantly positive correlation between C0 and TSH, FT3, and FT4. In addition, C0, FT4, and TSH all had significantly negative influence on newborn birth weight. Therefore, we evaluated the co-effects of C0 and THs on birth weight and found that the combinations of FT4 and C0 and of FT3 and C0 were significantly associated with birth weight, whereas that of TSH and C0 and that of TT4 and C0 were not.

Our study showed that FT4 and TSH had a significantly negative effect on neonate birth weight, whereas FT3 and TT4 had not. These results are consistent with that of Zhang et al., who found that higher TSH or FT4 concentrations throughout pregnancy were associated with lower birth weight (2). Other studies also showed that babies born to mothers with higher serum FT4 levels had an elevated risk for SGA, whereas those with lower serum FT4 levels had a higher risk for LGA (10, 11). Low FT4 levels, which may lead to an increase in circulating glucose, are associated with an increased risk of GDM, resulting in a higher placental glucose transport to the fetus and subsequent fetal weight gain (10, 12). Leon et al. also reported that subjects with low FT4 levels were significantly associated with a higher insulin resistance index, thus leading to an increased risk of LGA (12). Another potential mechanism is that higher TSH and FT4 levels accelerate the degradation of lipids and proteins, resulting in chronic energy deficiency in pregnant women, which has been shown to have a negative impact on neonate birth weight (2). FT4 is the active component of TT4, the most abundant TH in the body. Because the rate of conversion of TT4 to FT4 *in vivo* is limited by enzymes, there is no significant relationship between TT4 and birth weight. FT3 is three to five times more active than FT4. It is unclear whether maternal T3 actually crosses the placenta. FT3 is a TH that plays a direct biological role. Activation of deiodinase leads to a high rate of conversion of FT4 to FT3, resulting in low FT4 levels and high FT3 levels. High FT3 levels increase fetal weight directly through anabolic effects on fetal metabolism and stimulation of fetal oxygen consumption (9). A study showed that FT3 levels were positively associated with gestational weight gain in pregnant women (13). It has been reported that higher FT3 levels are associated with neonatal obesity, but the mechanism by which T3 affects fetal weight is unclear (9). Notably, THs are necessary for fetal cell differentiation and triggering organ development events in early pregnancy, and both high and low maternal FT4 levels were associated with adverse effects on birth weight (2). Korevaar et al. found an inverted U-shaped association of maternal FT4 with child IQ and gray matter volume (14).

The mitochondrial matrix enzyme, carnitine acetyltransferase, catalyzes the conversion of A-CoA and C0 to acetyl-carnitine and free Co-A, which plays a vital role in the production of energy in skeletal muscle, whereas TH is known to regulate several enzymes in this pathway (15). Maebashi et al. (16) reported that urinary carnitine excretion was positively correlated with serum thyroxine concentrations, with a significantly higher mean carnitine excretion in patients with hyperthyroidism and a lower carnitine excretion in patients with hypothyroidism compared with that in the control group. After correction of thyroid status, urinary carnitine excretion returned to normal in both groups (16). Another study examined total, free, and esterified carnitine levels in the skeletal muscle of patients with hyperthyroidism and hypothyroidism before and after drug treatment (17). A significant decrease was observed in total muscle carnitine concentrations in patients with hyperthyroidism compared with that in control subjects, which largely attributed to a decrease in the esterified carnitine portion. Total muscle carnitine levels were reduced in patients with hypothyroidism yet did not reach statistical significance, and no significant differences were found in esterified carnitine concentrations compared with control values. Meanwhile, Wong et al. (15) found that the level of acylcarnitine was relatively unremarkable in thyroid diseases. Other researchers argued that carnitine impairs the access of TH to the nucleus, thus decreasing the activity of TH (5, 18). An observational pilot study found that the symptoms of patients with subclinical hyperthyroidism relieved obviously after taking L-carnitine and selenium without any significant changes of their endocrine status (19). C0 was found to be negative on birth weight, and the combination of C0 and FT4 and of C0 and FT3 would significantly decrease the birth weight in this study, which reflected that C0 had a synergistic effect with THs, and assessment of total serum carnitine and changes in urinary carnitine excretion might help to find underlying mechanisms.

The present study has certain limitations. First, neonate birth weight could be influenced by many internal and external factors, including sex hormones, diet, medicine, and heredity. With so many confounding factors, subjects enrolled in this study could not be matched completely. Second, we did not assess the total serum carnitine and urinary carnitine, which might help explain the changing and transition of carnitine during pregnancy and the underlying mechanisms. We do find the co-effect of C0 and THs in the second trimester and their influence on neonate birth weight.

In conclusion, C0 and TH are of great importance in neonate birth weight, and routine examination of C0 and TH in the second trimester has a positive effect on the intervention of birth weight.

## Data availability statement

The original contributions presented in the study are included in the article/supplementary material. Further inquiries can be directed to the corresponding authors.

## Ethics statement

The studies involving human participants were reviewed and approved by IRB-20220254-R. The patients/participants provided their written informed consent to participate in this study. Written informed consent was obtained from the individual(s) for the publication of any potentially identifiable images or data included in this article.

## Author contributions

MY contributed to the collection, analysis, and interpretation of data as well as manuscript preparation. MS and QW contributed to the data collection and analysis, and YJ contributed to the interpretation of data. CJ contributed to the language editing. JX and QL contributed to the study design, data interpretation, and manuscript preparation. QL is the guarantor of this work and, as such, has full access to all the data in the study and takes responsibility for the integrity of the data and the accuracy of the data analysis. All authors contributed to the article and approved the submitted version.
